# Vitamin D deficiency status and its related risk factors during early pregnancy: a cross-sectional study of pregnant Minangkabau women, Indonesia

**DOI:** 10.1186/s12884-019-2341-4

**Published:** 2019-05-22

**Authors:** Arif Sabta Aji, E. Erwinda, Y. Yusrawati, Safarina G. Malik, Nur Indrawaty Lipoeto

**Affiliations:** 1 Postgraduate Biomedical Science Department, Faculty of Medicine, Andalas University, Padang, West Sumatra 25172 Indonesia; 2 Postgraduate Public Health Department, Faculty of Medicine, Andalas University, Padang, West Sumatra 25172 Indonesia; 3 Department of Obstetrics and Gynecology, Faculty of Medicine, Andalas University, Padang, West Sumatra 25172 Indonesia; 4 Eijkman Institute for Molecular Biology, Ministry of Research, Technology and Higher Education, Jakarta, 10430 Indonesia; 5 Nutrition Science Department, Faculty of Medicine, Andalas University, Padang, West Sumatra 25172 Indonesia

**Keywords:** Vitamin D, Vitamin D deficiency, Risk factors, Early pregnancy, Minangkabau women, West Sumatra

## Abstract

**Background:**

Vitamin D deficiency (VDD) is a common problem in reproductive-aged women and has become a major public health problem worldwide. The effect of VDD in pregnancy has been associated with several adverse pregnancy outcomes. This study aims to assess the serum levels of 25-hydroxyvitamin D (25(OH)D) in the first trimester and its associated factors (socio-demographics, pregnancy profiles, dietary intake, and maternal anthropometry measurements) for the determination of vitamin D deficiency status in early pregnancy.

**Methods:**

A cross-sectional study of 239 pregnant mothers in West Sumatra, Indonesia was conducted. We measured lifestyle, socio-demographics and pregnancy profile through a structured questionnaire and interview process. A semi quantitative-food frequency questionnaire (SQ-FFQ) was used to analyse the dietary intake of the pregnant women. Serum 25(OH)D concentrations were measured at < 13 weeks gestation using ELISA and logistic regression models were employed to identify the predictors of low vitamin D status.

**Results:**

The prevalence of first-trimester maternal VDD and sufficiency were 82.8 and 17.2% respectively. The median 25(OH)D was 13.15 ng/mL (3.00–49.29 ng/mL). The significant independent predictors were no working status (OR: 0.029;0.001–0.708) (*p* = 0.030); nulliparous parity status (OR: 7.634;1.550–37.608) (*p* = 0.012); length of outdoor activity status of less than an hour (OR: 9.659;1.883–49.550) (*p* = 0.007); and no consumption of supplements before pregnancy (OR: 4.49;1.081–18.563) (*p* = 0.039).

**Conclusions:**

The prevalence of VDD is common in early pregnancy among Minangkabau women. Recommendations and policies to detect and prevent such insufficiency during pregnancy should be developed by considering the associated factors.

## Background

Indonesia is a tropical country with abundant sun exposure, as it lies within the equatorial zone. However, low vitamin D status is still found in such countries Several studies on vitamin D status in pregnant women and women of childbearing age have been conducted and have shown that an average of more than 95% of individuals have low vitamin D status [1–3].

Vitamin D and parathyroid hormone are important in calcium homeostasis and bone mineralisation [4]. The main source of vitamin D is obtained by direct synthesis from sunlight to the skin and stimulation of pre-vitamin D_3_ development. Moreover, intake from diet and supplements will be synthesised as vitamin D_2_. However, dietary intake only provides 10% of vitamin D requirements as very few foods contain a high amount of vitamin D, so vitamin D supplements could be considered as a way of boosting such requirements [5]. Vitamin D_2_ and D_3_ are sequentially converted in the liver and kidneys into 25-hydroxyvitamin D, which is a major circulating form and is used to determine vitamin D status, and 1,25-dihydroxyvitamin D, the biologically active form respectively [6].

Vitamin D action during pregnancy is vital for foetal growth and development as it increases the calcium requirement to develop foetal bone growth. It also plays a role in helping the absorption of calcium in the digestive tract. Limited exposure to sunlight and food intake during pregnancy may cause insufficient body deposits in the foetus and mother. These factors affect the development and mineralisation of bone in the foetus and can have an adverse effect on the pregnancy outcomes. Several studies have reported the relationship between maternal VDD and adverse maternal and foetal outcomes, including gestational diabetes, pre-eclampsia, preterm labour, low birth weight, and caesarean section [7–10].

Personal lifestyle and cultural factors are important determinants for vitamin availability because of their influence on sun exposure and dietary intake [11, 12]. Determination of vitamin D status in the first trimester provides an opportunity to detect early vitamin D status and may help reduce pregnancy complications. The aim of this study is to assess serum 25(OH)D levels during early pregnancy and to determine the factors associated with low vitamin D status.

## Methods

### Study population

This was a cross-sectional study of maternal vitamin D status during early pregnancy in West Sumatra, Indonesia. The research location was divided into two different parts; urban/rural areas and mountainous/coastal areas. The study was conducted in five different cities between July and September 2017, the locations selected on the basis that they have the highest pregnancy rates for public health centres in the sub-districts of each district in West Sumatra. Pregnant women who visited a health care centre in each location were recruited with a total number of 239 women taking part. The inclusion criteria were as follows: 1) having received a pregnancy examination at a health centre in one of the research locations; 2) being in the first trimester of pregnancy (< 13 weeks); 3) being healthy as confirmed by a doctor (with no infections or history of communicable disease); and 4) being willing to comply with the study procedures by signing an informed consent form. The exclusion criteria comprised: 1) twin pregnancy; 2) having suffered or presently having a chronic disease history (e.g. diabetes, hypertension or heart attack); 3) anemia; 4) women routinely taking calcium supplements; 5) pregnant women taking medicine which would affect their vitamin D metabolism, such as antiepileptic agents, glucocorticoids, anti-estrogens or antiretroviral drugs; and 6) hypothyroidism. The selected subjects were interviewed and had a blood sample taken to measure their biochemical serum 25(OH)D level.

### Data collection

The data collected included the subject’s characteristics, anthropometric data, socio-economic status, blood pressure and dietary intake. Characteristic which included drug consumption history, maternal age, gravidity, parity, socioeconomic status, medical health history and lifestyle, were collected from personal interviews on enrolment on in the study. Anthropometric data, including weight, height, and the mid upper arm circumferences of the pregnant women, were measured by a midwife and trained enumerators. Body weight was measured to the nearest 100 g using an electronic scale (Seca 803, Seca GmbH. Co. kg, Hamburg, Germany) and height was measured to the nearest millimetre using a stadiometer (OneMed-Medicom stature meter, YF.05.05.V.A.1022, Jakarta, Indonesia) and mid-upper arm circumference was measured by using a meter line and rounded up to the nearest 0.1 cm (Medline-OneMed Medicom, Jakarta, Indonesia) in the more commonly used hand. Pre-pregnancy BMI was calculated by recalling the women’s pre-pregnancy weight by monitoring their longitudinal maternal and child book (KIA), classified according to World Health Organization guidelines for Asian populations (underweight, < 18.5 kg/m^2^; normal, 18.5–23.49 kg/m^2^; overweight, 23.5–24.99 kg/m^2^; pre-obese, 25–29.99 kg/m^2^; obese, ≥30 kg/m^2^) [13]. Gestational age was determined by the date of the last menstrual period and confirmed by ultrasound reports in the first trimester.

A lifestyle questionnaire was given to the participants, including questions about the duration of sun exposure, working status, physical activity, and sunscreen use. Sun exposure was calculated as an index of the hours per week the pregnant women spent outdoors exposed to sunlight, either during their leisure or working time. We then divided the exposure into two groups (≤1 h = inadequate or > 1 h = adequate). A midwife took systolic and diastolic blood pressure by three measurements before taking a blood sampling using an aneroid sphygmomanometer (OneMed-Medicom, Jakarta, Indonesia). Physical activity during the first trimester of pregnancy was measured using “The Global Physical Activity Questionnaire” (GPAQ), developed by the World Health Organization (WHO) [14]. The WHO STEPwise method was used to calculate physical activity and was expressed as Metabolic Equivalent minutes per day (METmins/day). The participants were classified as having “high activity” if they accumulated ≥3000 METmins/week, “moderate activity” if 3000 > MET≥600 or “low activity” if < 600 METmins/week.

### Dietary assessment

Dietary data were obtained from a validated semi quantitative food frequency questionnaire (SQ-FFQ) developed by Lipoeto (2004) [15]. Mean energy and nutrient intakes were calculated and compared with the Recommended Dietary Allowance (RDA) for pregnant women [16]. The SQ-FFQ was adapted to Minangkabau food habits; Minangkabau is an ethnic indigenous group in West Sumatra, Indonesia. The validated SQ-FFQ listed food fortified with vitamin D, natural food rich in vitamin D, and dietary supplements. More than 223 general food items containing potential sources of vitamin D in West Sumatra were included in the SQ-FFQ food list. Daily energy and nutritional intakes were calculated and compared with pregnant women RDA [14]. Based on calcium and vitamin D intake, the women were divided into two groups; those with an inadequate intake (< 15 μg/day or < 600 IU/day) and adequate intake (≥15 μg/day or ≥ 600 μg/day) of vitamin D. Furthermore, the participants were grouped based on those with an adequate intake (≥1300 mg/day) and inadequate intake (< 1300 mg/day) of calcium.

## Measurement of serum 25(OH)D

All the first trimester pregnant women enrolled in the study had blood samples taken from their antecubital vein. Non-fasting maternal blood samples were collected and banked by phlebotomists at the Biomedical Laboratory, Andalas University. 232 samples from the 239 participants were taken directly in public health centres in each research location. Subsequently, the samples were directly transferred and stored in the biomedical laboratory at the Faculty of Medicine, Andalas University, for serum 25(OH)D level assay. The serum samples were separated by centrifugation at 3500 rpm at 4 °C for 10 min, then stored in aliquots at − 80 °C. The serum 25(OH)D test was assessed using ELISA from Diagnostic Biochemistry Canada (DBC) 25-Hydroxyvitamin D (DBC, London, Ontario Canada). The inter-assay and intra-assay coefficients of variation of total serum 25(OH)D level were 5.0 and 8.10% at 21.87 ng/mL, and 2.4 and 9.9% at 45.01 ng/mL, respectively. There is no consensus on the optimal vitamin D level. In this study, vitamin D status was determined by 25-hydroxyvitamin D (25(OH)D) levels. We used the cut-off points suggested by the Institute of Medicine and vitamin D levels were categorised as sufficient (25(OH)D ≥ 20 ng/mL), insufficient (25(OH)D = 12–19 ng/mL), or deficient (25(OH)D < 12 ng/mL) [15]. 25(OH)D levels were the best marker for identifying vitamin D status and the major circulatingform of vitamin D.

### Statistical analyses

Data were presented as the mean levels of continuous variables as a mean ± SD (range), and numbers and percentages were used for the binary logistic of the binary and categorical data. The unit of measurement of vitamin D concentrations was standardised to the S.I. unit, ng/mL for 25-hydroxyvitamin D. Data analysis was performed to identify any differences in the data relating to baseline characteristics and dietary intake between the two groups. Furthermore, analysis of associated factors (socio-demographics, pregnancy profile, lifestyle, anthropometry and dietary intake) as predictors of VDD status was made using logistic regression.

The categorical data were analysed by a chi-squared test, and a student’s *t* test was used to compare the vitamin D serum levels of the two groups. Logistic regression models were used to estimate the OR’s of the dependent variable (vitamin D status) and the independent effects of known risk factors (e.g. lifestyle, dietary intake, socio-demographics, and pregnancy profile (data indicator for maternal health history, such as parity status, gestational age, maternal anthropometry, blood pressure and adverse pregnancy outcome)). All the data were managed and analysed descriptively using IBM SPSS, version 20.0, and presented as tables and Figs. A significance level of a *p* value of less than 0.05 with Odd Ratio (OR) and 95% CI was used to determine the relationship.

## Results

### Population characteristics

Table 1 shows the data characteristics of the first trimester of pregnant women, such as socio-demographics, pregnancy profile, and vitamin D intake. In total, 239 subjects were chosen, but only 232 successfully had their blood serum taken for analysis. Seven subjects failed to enroll in the study. The subjects completed the questionnaire and had anthropometric measurement and a blood test for vitamin D taken on their first visit for antenatal care in the public health centres. Their mean age was 29.77 ± 5.68 years, with most subjects in the > 30 years age group (45.30%). The geographical scope of the study was divided into coastal areas (40.90%) and mountainous areas (59.10%). Based on the working area, the study population comprised 48.70 and 51.30% from urban and rural areas respectively. Maternal education levels were 28.90% up to primary school, 40.50% to secondary school, and 30.60% from tertiary school level (diploma or high school). Women who were housewives and did not have an occupation were included in the no working group, while those who had jobs were included in the working group.

**Table 1:** Socio-demographic, pregnancy profile, and vitamin D intake of women (*N* = 232)

Independent variables	Percent (%)	Mean ± SD (range) / Median (IQR, 25, 75%)
*Socio-demographic factors*		
Location		
a. Padang	5.60	
b. Padang Pariaman	18.50	
c. Payakumbuh	26.30	
d. Lima Puluh Kota	32.80	
e. Pariaman	16.80	
Geographical status^a^		
a. Coastal	40.90	
b. Mountainous	59.10	
Urban status^b^		
a. Urban	48.70	
b. Rural	51.30	
Age (year)		29.77 ± 5.68 (min = 17, max = 44)
Age groups		
a. ≤20	3	
b. 21–25	23.70	
c. 26–30	28	
d. > 30	45.30	
Educational status		
a. Primary school	28.90	
b. Secondary school	40.50	
c. Tertiary school	30.60	
Working status		
a. Working	32.80	
b. Not working	67.20	
Household income per month (IDR)		IDR 2.400 (1.400) (min = IDR 700, max = IDR 48.000)
Household members		3 (1) (min = 4, max = 7)
*Pregnancy profiles*		
Gestational age (week)		10 (4) (min = 5, max = 12)
Parity status		
a. Nulliparous	25.40	
b. Multiparous	74.60	
Pre-pregnancy weight (kg)		55.48 ± 11.33 (min = 36, max = 95)
Height (cm)		154.35 ± 6.0 (min = 140, max = 176)
Pre-pregnancy BMI (kg/m^2^)		23.45 ± 4.56 (min = 14.10, max = 37)
Maternal weight of 1st trimester (kg)		56.15 ± 11.87 (min = 31, max = 93.9)
Upper arm circumference (cm)		27 ± 3.79 (min = 17, max = 38)
Smoking status		
a. Yes	2.60	
b. No	97.40	
Spontaneous abortion		
a. Yes	11.60	
b. No	88.40	
History of preterm birth		
a. Yes	3	
b. No	97	
Blood pressure (mmHg)		
a. Systolic		110.39 ± 11.32 (min = 90, max = 150)
b. Diastolic		75.41 ± 7.11 (min = 60, max = 90)
Factors related to vitamin D intake		
Consuming supplement before pregnancy		
a. Yes	12.90	
b. No	87.10	
Consuming supplement during 1st trimester		
a. Yes	38.40	
b. No	61.60	
Calcium and vitamin D supplements^a^		
a. Yes	16.40	
b. No	83.60	
Intake of vitamin D from food		5.32 (3.10) (min = 0.37, max = 23.63)
The level of vitamin D from food	3.40	
a. ≥15 mcg/day (adequate)	96.60	
b. < 15 mcg/day (inadequate)		
Intake of calcium from food		459.230 (367.87) (min = 353.27, max = 721.14)
The level of calcium from food		
a. ≥1300 mg/day (adequate)	9.90	
b. < 1300 mg/day (inadequate)	90.10	

^a^ Geographical status divided by research location in coastal area and mountainous area; ^b^ Urban status divided by location at urban area (Padang, Payakumbuh, and Pariaman) and rural area (Lima Puluh Kota and Padang Pariaman); BMI, body mass index

### Dietary, anthropometric, and pregnancy profile of the Minangkabau women

The median gestational age of the subjects on recruitment was 10 weeks (in the range 5–12 weeks). Approximately, 75% of them were and 25% nulliparous. The means of the maternal anthropometric values of the pregnant women were 55.48 ± 11.33 kg for pre-pregnancy bodyweight; 154.35 ± 6.0 cm for height; 23.45 ± 4.56 cm for pre-pregnancy BMI; 56.15 ± 11.87 kg for first trimester of bodyweight; and 27 ± 3.79 cm for mid-upper arm circumference (MUAC). Furthermore, with regard to dietary consumption and factors related to vitamin D intake in the study population, 80% of the women had not taken supplements before pregnancy, 62% were not taking supplements during pregnancy, and more than 90% had low vitamin D and calcium intake status. The median of vitamin D and calcium intake from food were 5.32 (3.1) mcg/d and 459.230 (367.87) mg/d for vitamin D and calcium intake, respectively. The dietary, anthropometric, and pregnancy profiles of the study population are summarised in Table 1.

### Serum 25(OH)D levels and Vitamin D status

Table 2. shows that median serum 25(OH)D levels were 13.15 ng/mL (in the range of 3.00–49.29 ng/mL). Most of the subjects had insufficient vitamin D status. 47% were vitamin D deficient, with serum levels lower than 12 ng/mL; 36.20% were vitamin D insufficient (concentration levels of serum between 12 and 19 ng/mL); and 17.20% had sufficient vitamin D status (concentration levels of serum above or equal to 20 ng/mL). Serum 25(OH)D level categories have varying reference guidelines. In this study, they were divided into three categories based on Institute of Medicine recommendations, with vitamin D levels categorised as sufficient (25(OH)D ≥ 20 ng/mL), insufficient (25(OH)D = 12–19 ng/mL) or deficient (25(OH)D < 12 ng/mL) [17]. For further multivariate analysis and meaningful statistical analysis, the subjects categorised as either vitamin D insufficient or deficient were combined into one category, with insufficiency-deficiency status (< 20 ng/mL) and with the sufficiency category being ≥20 ng/mL. 80.80% of the subjects had an insufficient-deficient vitamin D status, while 17.20% had a sufficient vitamin D status.

**Table 2:** Serum 25(OH)D levels among first trimester pregnant women (*N* = 232)

Variables	Percent (%)	Median (IQR, 25, 75%)
Serum 25(OH)D levels (ng/mL)		13.15 (9.98, min = 3.00, max = 49.29)
Vitamin D status		
a. Deficiency (< 12 ng/mL)	46.60	
b. Insufficiency (12–19 ng/mL)	36.20	
c. Sufficiency (≥20 ng/mL)	17.20	
Vitamin D status (dichotomous categorized)		
a. Deficiency-insufficiency (< 20 ng/mL)	82.80	
b. Sufficiency (≥20 ng/mL)	17.20	

### Lifestyle factors

Lifestyle factors in this study were successfully explored in the interview process at the recruitment stage and are shown in Table 3**.** The median of sunlight exposure duration was 60 min (in the range of 15–300 min); 47.80% of the subjects had less than an hour of sun exposure during the day, while 52.20% had more than 60 min. 70.30% used sunscreen and 29.70% did not to protect their skin from sun exposure. The occupation status of the subjects was as follows: 75.40% worked indoors and 24.60% worked outdoors. The style of dressing individuals included those who were covered, which means that they wore veils during their daily outdoor activities, and those who were uncovered, meaning they did not wear veils or cover their entire body surface when outdoors. The results of the study population reveal that 11.60 and 88.40% for < 27% uncovered and ≥ 27% uncovered group respectively. The physical activity levels in the first trimester were 39.70% low level, 35.80% moderate and 24.60% high physical activity level (PAL).

**Table 3:** Lifestyle factors of first trimester pregnant women (*N* = 232)

Independent variables	Percent (%)	Median (IQR 25, 75%)
Sunlight exposure (minute/day)		60 (53.75 (min = 15, max = 300)
Length of outdoor activity		
a. < 60 min/week	47.80	
b. ≥60 min/week	52.20	
Sunscreen application		
a. Yes	70.30	
b. No	29.70	
Occupation		
a. Indoor	75.40	
b. Outdoor	24.60	
Dressing style		
a. < 27% uncovered	11.60	
b. ≥27% uncovered	88.40	
Physical activity		
a. Low	39.70	
b. Moderate	35.80	
c. High	24.60	

### Factors associated with VDD

Univariate analysis of the association between potential associated factors and vitamin D deficiency-insufficiency status during early pregnancy are shown in Table 4. When using univariate binary logistic regression analysis, the vitamin D sufficient group (25(OH)D ≥ 20 ng/mL) was shown to be older; with a higher household income per month; lower bodyweight in the first trimester and before pregnancy; lower education levels; lower mid-upper arm circumference; lower pre-pregnancy BMI status in the overweight and obese group; represent a lower proportions of nulliparous pregnant women; had a lower level of sunscreen application; and more outdoor activity hours compared to the vitamin D deficiency-insufficiency group (25(OH)D < 20 ng/mL). A higher proportion of pregnant women who lived in coastal and rural areas had vitamin D sufficiency status. However, there were no significant differences between those variables and vitamin D status during early pregnancy in the Minangkabau pregnant women.

**Table 4:** Factors associated with low vitamin D status

Variables		OR (95% CI)		*P* value
Age (year)		1.020	0.862–1.208	0.814
Age group	< 20	1.00		
	21–25	0.777	0.060–10,143	0.848
	26–30	0.172	0.009–3.279	0.242
	> 30	0.189	0.040–111.259	0.360
Education levels	Primary	1.00		
	Secondary	0.568	0.182–1.775	0.330
	Tertiary	0.429	0.100–1.829	0.253
Working status	Working	1.00		0.030
	Not Working	0.029	0.001–0.708	
Household income/month (IDR)		1.00	1.00–1.00	0.681
Urban status	Urban	1.00		0.882
	Rural	1.109	0.285–4.312	
Geographical status	Coastal	1.00		
	Mountainous	0.424	0.121–1.486	0.180
Gestational age (week)		0.945	0.771–1.157	0.583
Parity status	Multiparous Nulliparous	1.00 7.634	1.550–37.608	0.012
Bodyweight of 1st trimester (kg)		0.975	0.826–1.151	0.764
Upper arm circumference (cm)		1.118	0.882–1.416	0.358
Pre-pregnancy bodyweight (kg)		1.007	0.840–1.207	0.939
Pre-pregnancy BMI (kg/m^2^)		0.920	0.644–1.315	0.647
Pre-pregnancy BMI status	Underweight	1.00		
	Normal	0.664	0.118–3.731	0.642
	Overweight	0.101	0.003–2.977	0.184
	Pre-obese	0.990	0.038–26.028	0.995
	Obese	2.423	0.025–232.393	0.704
Blood pressure (mmHg)	Systolic	1.00	0.947–1.056	0.994
	Diastolic	0.971	0.882–1.070	0.552
Sunscreen application	No	1.00		0.810
	Yes	1.147	0.375–3/507	
Type occupation	Indoor	1.00		0.081
	outdoor	17.713	0.704–445.491	
Dressing style	< 27% uncovered	1.00	0.267–5.552	0.799
	≥27% uncovered	1.218		
Physical activity level	Low-moderate	1.00		0.731
	High	0.815	0.255–2.610	
Outdoor activity		0986	0.972–1.001	0.070
The length of outdoor activity	≥60 min/week	1.00		0.007
	< 60 min/week	9.659	1.883–49.550	
Vitamin D intake (mcg/d)		0.988	0.885–1.103	0.835
Calcium intake (mg/d)		1.00	0.999–1.000	0.269
Vitamin D intake status	Inadequate	1.00	0.222–188.964	0.277
	Adequate	6.483		
Calcium intake status	Inadequate	1.00	0.086–9.904	0.946
	Adequate	0.921		
Consuming supplement before pregnancy	Yes	1.00	1.081–18.563	0.039
	No	4.49		
Consuming supplement during 1st trimester	No	1.00	0.172–1.443	0.199
	Yes	0.499		
Consuming vitamin D and calcium supplement	No	1.00	0.259–4.289	0.940
	Yes	1.056		

*OR* odds ratio, *CI* confidence interval, *Ref* reference category

Bold number is indicated *P* < 0.05

Serum 25(OH)D < 20 ng/mL (*n* = 192) and serum ≥20 ng/mL (*n* = 40)

1.00 as a reference group

A significant association was shown between all the risk factor variables and low vitamin D status after performing a binary logistic regression. The results show that four risk factors were significantly associated with low vitamin D status; non working status (OR: 0.029, CI 95% 0.001–0.708) (*p* value = 0.030); Nulliparous parity status (OR: 7.634, CI 95% 1.550–37.608) (*p* value = 0.012); length of outdoor activity status of less than an hour (OR: 9.659, CI 95% 1.883–49.550) (*p* value = 0.007); and not taking supplements before pregnancy (OR: 4.49, CI 95% 1.081–18.563) (*p* value = 0.039). No significant associations were observed between any other factors and vitamin D deficiency-insufficiency status.

## Discussion

This study has reported the prevalence of low maternal vitamin D status in early pregnancy of 10 weeks (in the range of 5–12 weeks). The subjects were obtained by approaching singleton pregnant women at Public Health Centres in the six districts of West Sumatra Province, Indonesia. Only healthy pregnant women were included, ones Who did not have any pre-existing hypertension, diabetes or other medical issues that could have increased their risk of developing pregnancy complications or VDD. The majority of our study population were either vitamin D deficient or insufficient (82.80%, < 20 ng/mL), compare to those who were vitamin D sufficient (17.20%, ≥20 ng/mL) according to the criteria published by the Institute of Medicine [17]. On the other hands, the Endocrine Society published different cut-offs for vitamin D status, and reported that women who had less than 10 ng/mL were in the severe deficiency group; those who had less than 20 ng/mL were in the deficiency group; those between 30 and 44 ng/mL were in the sufficiency group; and those above 100 ng/mL were in the toxicity group [18]. Using the Endocrine Society cut-offs in our study population would change the prevalence of VDD to 36.20% for severe deficiency, 46.60% for deficiency and 14.70% for insufficiency, with only 2.60% having vitamin D sufficiency status.

Indonesia has a prevalence of VDD status among women of childbearing age, children, and pregnant women. Recent studies in North Sumatra have shown that 70% of women of childbearing age (*n* = 100) had deficient status, while 29 and 1% had insufficient and sufficient status, respectively [19]. A survey of children aged from 0.5 to 12 years in the South East Asian Nutrition Surveys (SEANUTS), based on a total of 16.744 children from Indonesia, Malaysia, Thailand, and Vietnam, revealed that the percentage of children with adequate 25(OH)D (≥75 nmol/L equal to ≥30 ng/mL) was as low as 5% (in Indonesia) and up to 20% (in Vietnam). Vitamin D insufficiency (< 50 nmol/L equal to < 20 ng/mL) was observed in between 40 to 50% of the children in all these countries [20]. Bardosono et al., [2] reported that among 143 healthy pregnant women on their first visit to maternal clinics in Jakarta, Indonesia, 25.20% were found to be deficient in calcium and 90.20% vitamin D. This high prevalence of VDD was due to gender, childhood consequences, type of occupation, physical activity, vitamin D intake, body fat percentage, duration of sun exposure, living in an urban area, region of residence within the country, and religion, which all significantly increased the odds of being vitamin D insufficient [2, 19, 20].

Low serum 25(OH)D levels in our study population are most likely due to the subjects being in their first trimester of pregnancy, therefore a greater proportion of them would be suffering from morning sickness, resulting in limited outdoor physical activity, and very few women take multivitamin supplements. Bukhary et al., [21] reported that 90% of first trimester pregnant women studied were suffering from hypovitaminosis D. Jan Mohammed et al., [22] conducted a study in different terms of pregnancy, and found that the prevalence of low vitamin D status (< 30 ng/mL) among second and third trimester women was 60 and 37%, respectively. A study measuring the vitamin D level of 200 women in India also mentioned similar findings, where the prevalence of VDD was significantly higher in the third trimester, than in the first and second trimester [23]. The reduced prevalence of VDD during pregnancy might be due to a significant increase in dietary intake and supplied multivitamins during pregnancy [22].

In this study, few subjects took supplements before pregnancy. There was an association between low consumption of supplements and vitamin D status (*p* = 0.039). Pregnant women who did not take supplements before pregnancy had a 4.5 times increased risk of VDD (OR: 4.49, CI 95% 1.081–18.563) (*p* value = 0.039). Having a proper nutritional status through dietary supplementation before pregnancy enhances prenatal health, prepares nutrition reserves in the preconception period, and prevents the risk of adverse pregnancy and foetal outcomes associated with nutrient deficiencies [24]. Furthermore, our subjects also had a lower vitamin D and calcium intake (5.23 mcg/d (3.10, range 0.37–23.63 mcg/d) vs 459.230 mg/d (367.87, range 353.27–721.14). The results with regard to vitamin D intake in our study population shows that the level is almost half the Recommended Intake of 10 mcg/day proposed by the Institute of Medicine (IOM) and Indonesia Recommended Dietary Allowance (RDA) [16, 17]. Even though diet and supplement intake only provide low levels of vitamin D, low vitamin D intake status is considered to be one of the risk factors in VDD. A study by Bukhary et al. showed that dietary intake of vitamin D was a significant predictive factor, together with ethnic group, educational status and sun protection [21]. The first trimester of pregnancy is associated with multiple instances of morning sickness. This condition leads to poor dietary intake during this period and worsens if it means women are unable to achieve a balanced nutrition, which is an important factor in fulfilling nutrient requirements. Increasing dietary consumption of vitamin D, such as milk and other dairy products, and the use of multivitamins and dietary supplements may be needed, and these solutions might help to maintain a sufficient vitamin D level [25]. Recent clinical guidelines report that taking more than 600 IU/day of vitamin supplements may increase and maintain vitamin D levels at levels higher than 30 ng/mL (75 nmol/L), but further research is needed to determine the appropriate dosage of supplements [26–29].

There are several possible reasons for decreasing 25(OH)D serum levels; not only low consumption of vitamin D-rich food, but also certain external factors that contribute to preventing bodies from being exposed to sunlight. However, sources of vitamin D from food are lower and only contributes 10% of total needs. The main source of vitamin D is exposure to UVB sunlight, which contributes to 90% of vitamin D requirements [30]. Therefore, the major cause of VDD is inadequate sunlight exposure [31]. Indonesia is a tropical country with only two season per year and we conducted this study in the dry season at that time. In this current study, we did the data collection during July–September 2017 in West Sumatra with the daily hours of sunshine averagely about 5 h [32]. This makes Sumatra has longer sunshine hours compared to other months and those data might influence our result of the study. A positive correlation was found between sun exposure and vitamin D status. Most of the women who had less exposure to sunlight were vitamin D deficient. Pregnant women who are less physically active or have a sedentary indoor lifestyle have less exposure to sunlight [23, 33]. In this study it was revealed that women who had an outdoor activity status of less than an hour had a tenfold increase in the risk of developing VDD (OR: 9.659, CI 95% 1.883–49.550) (*p* value = 0.007). Our previous study on the third trimester pregnant women also reported that those who living in mountainous areas, worked indoors, or who were engaged in low-middle physical activity had a greater risk of developing VDD [11]. Applying sunscreen with a high sun protection factor could reduce vitamin D synthesis in the skin by more than 95%. Many factors influence the vitamin D status of individuals and populations, including latitude, season, time spent outdoors, clothing habitually worn, sunscreen use, weight status, skin colour, and some medications and medical conditions [34].

Pregnant women with nulliparous parity status tend to have higher serum 25(OH)D levels than multiparous women [35]. However, different studies related to serum 25(OH)D levels and parity have presented conflicting results [36–38]. Our study population, who were nulliparous women, were likely to have lower 25(OH)D serum levels than their multiparous counterparts with levels of 12.70 ng/mL and 14.45 ng/mL respectively. After adjusting for multivariate logistic regression, parity status was included as one of the risk factors in VDD. Pregnant women with nulliparous parity status had an eight times higher risk of developing VDD (OR: 7.634, CI 95% 1.550–37.608) (*p* value = 0.012). This finding is similar to that of Perez et al., who examined first trimester vitamin D status and the factors associated with the lower 25(OH)D levels. Nulliparous women (OR: 2.47;*p* = 0.002), those of non-Caucasian ethnicity (OR: 36.29;*p* = 0.001), and the season (OR: 10.93;p = 0.001) at the time of sampling were factors related to deficient 25(OH)D levels [36]. Different results were obtained by Choi et al., and Ates et al., who reported that parity status was not associated with serum 25(OH)D levels during the first trimester of pregnancy [39, 40]. However, nulliparous pregnant women have a greater risk of certain pregnancy complications such as pre-eclampsia and preterm delivery compared to multiparous women [25].

The limitations of this study include possible bias due to the small sample size and the self-selection of study subjects, and the fact that a gold-standard method for measuring 25(OH)D levels was not used. In addition, we could not completely evaluate underlying factors such as pigmentation, physical activity levels, macronutrient values, sun protection score, and the length of sun exposure which is during the dry season, Sumatra has more sunshine hours compared to other months. These data might influence our result of study. Recall bias from questionnaires is a common problem when conducting an observational study, as the data are obtained through an interview session. However, we tried to reduce bias by recruiting a trained interviewer who was a nutritionist to collect the dietary assessments and other questionnaires. The information about the background of the study population (socioeconomic and sociodemographic) used in the study was collected from accurate and comprehensive databases from the maternal care units in the public health centres in each district. The prevalence of VDD status in the first trimester may not be compared to other population studies with different geographical characterisation. However, the main strength of this study is the involvement of biochemical and dietary intake measurements of vitamin D in the West Sumatran population. External factors were examined to determine predictive factors in developing VDD status, such as lifestyle, factors related to vitamin D intake, and pregnancy profiles. A larger sample is needed in future studies, especially from the Indonesian population, and it would be better if subjects from different areas of the country were recruited, in order to form a better picture of vitamin D status during pregnancy in Indonesia.

## Conclusions

Despite living in a country in Southeast Asia with high sun exposure, pregnant Minangkabau women reported a high prevalence of first trimester VDD, which was related to not working, nulliparous parity, partaking in outdoor activities for less than one hour per day, and not consuming vitamin D supplements before pregnancy. Recommendations and policies to detect and prevent insufficiency of vitamin D during pregnancy should be developed with consideration of the associated factors.

## Abbreviations

25(OH)D: 25-hydroxyvitamin D
BMI: Body mass index
CI: Confidence interval
ELISA: Enzyme-linked immunosorbent assay
IOM: Institute of Medicine
MUAC: Mid-upper arm circumference
PAL: Physical activity levels
RDA: Recommended dietary allowance
SQ-FFQ: Semi quantitative food frequency questionnaire
VDD: Vitamin D deficiency

## References

[1] Wibowo N, Bardosono S, Irwinda R, Syafitri I, Putri AS, Prameswari N (2017). Assessment of the nutrient intake and micronutrient status in the first trimester of pregnant women in Jakarta. Med J Indonesia.

[2] Bardosono S (2016). Maternal micronutrient deficiency during the first trimester among Indonesian pregnant women living in Jakarta. JKI.

[3] Sari DK, Damanik HA, Lipoeto NI, Lubis Z (2013). Is micro evolution in tropical country women resulting low 25(OH)D level?: a cross sectional study in Indonesia. J Nutr Food Sci.

[4] Triunfo S, Lanzone A (2016). Potential impact of maternal vitamin D status on obstetric well-being. J Endocrinol Investig.

[5] Binkley N, Ramamurthy R, Krueger D (2010). Low Vitamin D status: definition, prevalence, consequences and correction. Endocrinol Metab Clin N Am.

[6] Bikle D, Vitamin D, De Groot LJ, Chrousos G, Dungan K, Feingold KR, Grossman A, Hershman JM (2000). Production, Metabolism, and Mechanisms of Action. Endotext.

[7] Bodnar LM, Simhan HN, Catov JM, Roberts JM, Platt RW, Diesel JC (2014). Maternal vitamin D status and the risk of mild and severe preeclampsia. Epidemiology.

[8] Dror DK (2011). Vitamin D status during pregnancy: maternal, fetal, and postnatal outcomes. Curr Opin Obstet Gynecol.

[9] Scholl TO, Chen X, Stein P (2012). Maternal Vitamin D status and delivery by cesarean. Nutrients.

[10] Khalessi N, Kalani M, Araghi M, Farahani Z (2015). The relationship between maternal Vitamin D deficiency and low birth weight neonates. J Family Reprod Health.

[11] Aji AS, Desmawati D, Yerizel E, Lipoeto NI (2018). The association between lifestyle and maternal vitamin D levels during pregnancy in West Sumatra. Asia Pac J Clin Nutr.

[12] Nimitphong H, Holick MF (2013). Vitamin D status and sun exposure in Southeast Asia. Dermatoendocrinol.

[13] Expert Consultation WHO (2004). Appropriate body-mass index for Asian populations and its implications for policy and intervention strategies. Lancet.

[14] Armstrong T, Bull F (2006). Development of the world health organization global physical activity questionnaire (GPAQ). J Public Health.

[15] Lipoeto NI, Agus Z, Oenzil F, Wahlqvist M, Wattanapenpaiboon N (2004). Dietary intake and the risk of coronary heart disease among the coconut-consuming Minangkabau in West Sumatra. Indonesia Asia Pac J Clin Nutr.

[16] Ministry of Health Republic of Indonesia (2013). Dietary intake reference in Indonesia. Jakarta: Ministry of Health republic of Indonesia.

[17] Institute of Medicine (2011). Dietary reference intakes for calcium and Vitamin D.

[18] Holick MF, Binkley NC, Bischoff-Ferrari HA, Gordon CM, Hanley DA, Heaney RP (2011). Evaluation, treatment, and prevention of vitamin D deficiency: an Endocrine Society clinical practice guideline. J Clin Endocrinol Metab.

[19] Sari DK, Damanik HA, Lipoeto NI, Lubis Z (2013). Low serum 25(OH)D levels are associated with single nucleotide polymorphisms of the vitamin D receptor gene and lifestyle factors, especially in women with higher body fat percentage. Obes Res Clin Pract.

[20] Poh BK, Rojroongwasinkul N, Nguyen BKL, null S, Ruzita AT, Yamborisut U (2016). 25-hydroxy-vitamin D demography and the risk of vitamin D insufficiency in the south east Asian nutrition surveys (SEANUTS). Asia Pac J Clin Nutr.

[21] Bukhary NBI, Isa ZM, Shamsuddin K, Lin KG, Mahdy ZA, Hassan H, et al. Risk factors for antenatal hypovitaminosis D in an urban district in Malaysia. BMC Pregnancy Childbirth. 2016;16. 10.1186/s12884-016-0939-3. 10.1186/s12884-016-0939-3 PMC4944244 27411716

[22] Jan Mohamed HJ, Rowan A, Fong B, Loy S-L. Maternal serum and breast Milk Vitamin D levels: findings from the Universiti Sains Malaysia pregnancy cohort study. PLoS One. 2014;9. 10.1371/journal.pone.0100705. 10.1371/journal.pone.0100705 PMC4081124 24992199

[23] Singh D (2016). Vitamin D status in pregnant women of Udaipur. Biochem Anal Biochem.

[24] World Health Organization (2016). WHO recommendations on antenatal Care for a Positive Pregnancy Experience.

[25] Flood-Nichols SK, Tinnemore D, Huang RR, Napolitano PG, Ippolito DL. Vitamin D deficiency in early pregnancy. PLoS One. 2015;10. 10.1371/journal.pone.0123763. 10.1371/journal.pone.0123763 PMC4405493 25898021

[26] Sari DK. Vitamin D supplementation increased 25(OH)D serum levels but did not reach normal range in north Sumatera women with vitamin D receptor gene polymorphism. Innovations in Nutrition, Food Sciences and Public Health, JAPAN. J Nutr Food Sci. 2017;7:4(Suppl). 10.4172/2155-9600-C1-044.

[27] Mazahery H, von Hurst PR (2015). Factors affecting 25-Hydroxyvitamin D concentration in response to Vitamin D supplementation. Nutrients.

[28] Mithal A, Kalra S (2014). Vitamin D supplementation in pregnancy. Indian J Endocrinol Metab.

[29] Harvey NC, Holroyd C, Ntani G, Javaid K, Cooper P, Moon R (2014). Vitamin D supplementation in pregnancy: a systematic review. Health Technol Assess.

[30] Björn LO, Wang T (2000). Vitamin D in an ecological context. Int J Circumpolar Health.

[31] Holick MF, Chen TC (2008). Vitamin D deficiency: a worldwide problem with health consequences. Am J Clin Nutr.

[32] BMKG Indonesia. Prakiraan Musim Kemarau 2017 di Indonesia | BMKG. BMKG | Badan Meteorologi, Klimatologi, dan Geofisika. 2017. https://www.bmkg.go.id/iklim/prakiraan-musim.bmkg?p=prakiraan-musim-kemarau-2017-di-indonesia&tag=prakiraan-musim&lang=ID. (Accessed 9 Aug 2018).

[33] Sari DK, Damanik HA, Lipoeto NI, Lubis Z. Is micro evolution in tropical country women resulting low 25(OH)D level?: a cross sectional study in Indonesia. J Nutr Food Sci. 2013;4. 10.4172/2155-9600.1000246.

[34] Rockwell M, Kraak V, Hulver M, Epling J. Clinical Management of low Vitamin D: a scoping review of physicians’ practices. Nutrients. 2018;10. 10.3390/nu10040493. 10.3390/nu10040493 PMC5946278 29659534

[35] Zhao X, Fang R, Yu R, Chen D, Zhao J, Xiao J. Maternal Vitamin D status in the late second trimester and the risk of severe preeclampsia in southeastern China. Nutrients. 2017;9. 10.3390/nu9020138. 10.3390/nu9020138 PMC5331569 28216561

[36] Pérez-López FR, Fernández-Alonso AM, Ferrando-Marco P, González-Salmerón MD, Dionis-Sánchez EC, Fiol-Ruiz G (2011). First trimester serum 25-hydroxyvitamin D status and factors related to lower levels in gravids living in the Spanish Mediterranean coast. Reprod Sci.

[37] Makgoba M, Nelson SM, Savvidou M, Messow C-M, Nicolaides K, Sattar N (2011). First-trimester circulating 25-Hydroxyvitamin D levels and development of gestational diabetes mellitus. Diabetes Care.

[38] Andersen LB, Abrahamsen B, Dalgård C, Kyhl HB, Beck-Nielsen SS, Frost-Nielsen M (2013). Parity and tanned white skin as novel predictors of vitamin D status in early pregnancy: a population-based cohort study. Clin Endocrinol.

[39] Choi R, Kim S, Yoo H, Cho YY, Kim SW, Chung JH (2015). High prevalence of Vitamin D deficiency in pregnant Korean women: the first trimester and the winter season as risk factors for Vitamin D deficiency. Nutrients.

[40] Ates S, Sevket O, Ozcan P, Ozkal F, Kaya MO, Dane B (2016). Vitamin D status in the first-trimester: effects of Vitamin D deficiency on pregnancy outcomes. Afr Health Sci.

